# Photocatalytic Mechanisms for Peroxymonosulfate Activation through the Removal of Methylene Blue: A Case Study

**DOI:** 10.3390/ijerph16020198

**Published:** 2019-01-11

**Authors:** Jorge Rodríguez-Chueca, Esther Alonso, Devendra Narain Singh

**Affiliations:** 1 Department of Industrial Chemical & Environmental Engineering, Escuela Técnica Superior de Ingenieros Industriales, Universidad Politécnica de Madrid, Calle José Gutiérrez Abascal 2, 28006 Madrid, Spain; e.alazaro@alumnos.upm.es; 2 Department of Civil Engineering, Indian Institute of Technology Bombay, Powai, Mumbai-400076, India; dns@civil.iitb.ac.in

**Keywords:** sulfate radicals, peroxymonosulfate, iron activation, TiO_2_ activation, dye, synergistic effect

## Abstract

Industrial activity is one of the most important sources of water pollution. Yearly, tons of non-biodegradable organic pollutants are discharged, at the least, to wastewater treatment plants. However, biological conventional treatments are unable to degrade them. This research assesses the efficiency of photocatalytic activation of peroxymonosulfate (PMS) by two different iron species (FeSO_4_ and Fe^3+^-citrate) and TiO_2_. These substances accelerate methylene blue removal by the generation of hydroxyl and sulfate radicals. The required pH and molar ratios PMS:Fe are crucial variables in treatment optimization. The kinetic removal is reduced by the appearance of scavenger reactions in acidic and basic conditions, as well as by the excess of PMS or iron. The best performance is achieved using an Fe^3+^-citrate as an iron catalyst, reaching the total removal of methylene blue after 15 min of reaction, with a molar ratio of 3.25:1 (1.62 mM of PMS and 0.5 mM Fe^3+^-citrate). Fe^3+^-citrate reached higher methylene blue removal than Fe^2+^ as a consequence of the photolysis of Fe^3+^-citrate. This photolysis generates H_2_O_2_ and a superoxide radical, which together with hydroxyl and sulfate radicals from PMS activation attack methylene blue, degrading it twice as fast as Fe^2+^ (0.092 min^−1^ with Fe^2+^ and 0.188 min^−1^ with Fe^3+^-citrate). On the other hand, a synergistic effect between PMS and titanium dioxide (TiO_2_) was observed (*S_PMS/TiO2/UV-A_* = 1.79). This synergistic effect is a consequence of PMS activation by reaction with the free electron on the surface of TiO_2_. No differences were observed by changing the molar ratio (1.04:1; 0.26:1 and 0.064:1 PMS:TiO_2_), reaching total removal of methylene blue after 80 min of reaction.

## 1. Introduction

All the human activities require the use of water, either directly or indirectly. The different human uses of water contaminate it with diverse chemical and biological substances. Water pollution generates environmental, economic, social, and health problems [1,2]. For that, it is compulsory to restore the water quality. For example, industrial activities generate extremely large amounts of organic pollutants—in many cases, recalcitrant organics that only can be removed by chemical oxidation treatments [3]. This is the case, for instance, of some dyes that are not only used in the textile industry, but also for other applications, as the case of methylene blue used in medicine and aquaculture [4]. Nevertheless, traditional chemical oxidation treatments are based on the use of chlorine or ozone as oxidants, with the main disadvantage being the generation of by-products such as trihalomethanes, haloacetic acids, or bromates [5]. Adsorption, sedimentation, and chemical precipitation are other technologies that can remove recalcitrant organics, along with the advanced oxidation processes (AOPs), having been investigated over the last decades [4,6,7,8].

There is a wide range of AOP technologies; the most studied are related to the use of photocatalysts as TiO_2_ [9,10], the Fenton’s reagent [11,12], or combination of iron species with H_2_O_2_, which unchains in both cases to generate highly oxidant free radicals (mainly hydroxyl radicals). However, during the last years, the scientific community has paid attention to AOPs based on the generation of sulfate radicals. This interest is reflected in the increase of publications in literature [13]. Sulfate radical-based advanced oxidation processes (SR-AOPs) have as a special feature the generation of not only sulfate radicals, but also hydroxyl radicals, depending on the operating conditions. Therefore, this consortium of radicals enhances the efficiency of hydroxyl radical-based advanced oxidation processes (HR-AOPs), such as photo-Fenton, for instance [14].

SR-AOPs can be performed through the previous activation of peroxymonosulfate (PMS) or persulfate (PS). This activation is required because of the low oxidation potential of the substances by themselves. The main activation techniques are (i) thermal, (ii) UV-radiation, and (iii) transition metals or metal oxides as catalysts [13,14]. These are the most common activation methods, but there are others that are less explored—for instance, the work at alkaline conditions or the use of carbonaceous-based materials [13]. Iron, cobalt, or nickel have been reported in literature as good activators of PMS and PS [15]. However, a lower combination of these oxidants with titanium dioxide (TiO_2_) is reported. TiO_2_ is the most studied semiconductor photocatalyst for water purification. Some authors have explored the activation of PMS by TiO_2_ [16,17]. It seems that a synergistic effect occurs when both substances are coupled: TiO_2_ decomposes PMS molecules, increasing the radical generation rate, and the photolysis of PMS can enhance the reactivity of TiO_2_ and inhibit the recombination of the electron–hole pair through trapping photo-induced electrons [18,19].

The main goal of this research is to study the activation of PMS through different iron species (FeSO_4_ and Fe^3+^-citrate) and TiO_2_ in the presence of UV-A radiation. The efficiency of the treatment was assessed through the discoloration of methylene blue dissolved in water.

## 2. Materials and Methods

### 2.1. Reagents

The methylene blue (MB; C_16_H_18_ClN_3_S; *M*_w_ = 319.8 g/mol) was provided by Scharlau (Spain) and used as received, without further purification. The UV-visible spectrum of MB consists of a main characteristic absorption band at 660 nm.

Discolouration treatments were carried out using potassium peroxymonosulfate (2KHSO_5_·KHSO_4_·K_2_SO_4_, PMS, Merck) as an oxidant. Iron (II) sulfate heptahydrate (FeSO_4_·7H_2_O, Panreac), iron (III)-citrate (C_6_H_5_FeO_7_; ACROS Organics) and titanium dioxide (Evonik Aeroxide^®^ P25) were assessed as catalytic activators of PMS. Besides, CaCO_3_ (Scharlau) was used to scavenge hydroxyl radicals, while HCl and NaOH (Scharlau) was used to adjust pH.

### 2.2. Experimental Setup

All experiments were carried out in batch mode in a lab-scale reactor illuminated by a black light lamp (Philips TL 6 W; maximum emission peak at 365 nm). The reactor has a volume capacity of 200 mL, with 1 cm depth of the solution, and 15 cm between the UV-A lamp and the MB solution. These parameters allow the optimal removal of MB while avoiding shadow zones, and to obtain a totally stirred reactor. This is required to obtain an optimal removal of MB.

### 2.3. Experimental Procedure

Samples were prepared by dissolving 10 mg/L of MB in deionised form. Then, depending on the type of treatment, different dosages (0.81–3.25 mM) of PMS were added and combined with different dosages (0.1 to 1 mM) of iron activators (FeSO_4_·7H_2_O and Fe^3+^-citrate) or TiO_2_ (1.25–5.00 mM). The reagents were directly added to the reactor at the beginning of each experiment, when the UV-A radiation was switched on. Treatments were applied for a maximum of 120 min at a neutral pH (pH = 7), as well as at pH 4 and 11 to study the effect of pH on the removal efficiency.

Samples of the MB solution were collected at periodic intervals during the reaction, and analysed using a Mettler Toledo UV5 spectrophotometer. The discolouration of MB was obtained by measuring the absorbance at maximum wavelength (λ_max_ = 660 nm) and by computing the concentration from the calibration curve. The temperature and pH of the samples were monitored using a pH Meter from XS Instruments (model PC 8).

## 3. Results and Discussion

### 3.1. Photolytic Activation of Peroxymonosulfate and TiO_2_ as a Benchmark

**Figure 1:**
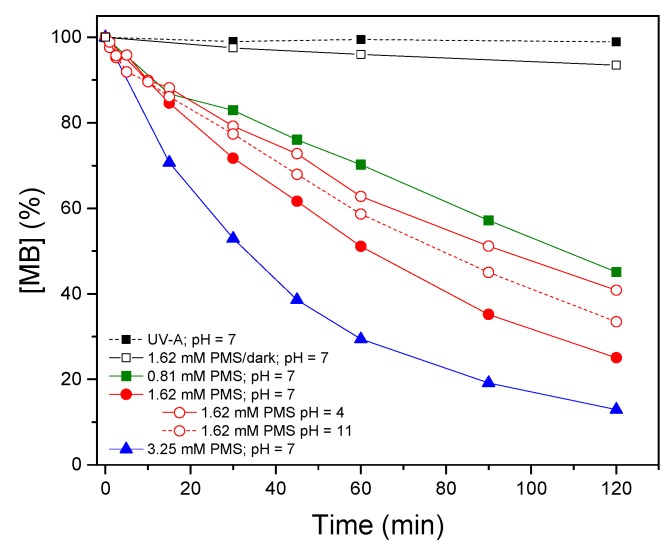
Influence of peroxymonosulfate (PMS) dosage (0.81, 1.62 and 3.25 mM), and pH (4, 7 and 11) in methylene blue (MB) discolouration, through PMS photoactivation with UV-A radiation.

As mentioned in the goal of the manuscript, this research tries to understand the pathways through which PMS is photocatalytically activated, either by Fe species or TiO_2_. However, it is required to establish the benchmark to allow later comparisons. This benchmark corresponds to UV-A photolytic activation of PMS represented in the Figure 1.

As observed in Figure 1, PMS/UV-A promotes the removal of MB by increasing the added dose of oxidant. After 120 min, 87 and 75% of MB was removed, with 3.25 and 1.62 mM respectively of PMS at pH ≈ 7. Because of the small differences between dosages, and the supposed drawbacks to using a higher dose, 1.62 mM of PMS was selected for the following treatments. Although previous research has shown a non-pH-dependency of PMS [15], it was found that efficiency changes during acidic and basic conditions (pH = 4 and 11). The results confirm that no large differences are observed in the efficiency of MB removal with pH. In any case, the best performance is reached at a neutral pH. This fact is important to note, because the possibility of treating wastewater at a neutral pH means a reduction in the operating costs as consequence of a previous acidification or basification, and later neutralization before the discharge to receive water bodies. However, although there is a pH dependency, these treatments could be applied under acidic or basic conditions with remarkable efficiencies. This clearly contrasts with other AOPs that are highly dependent on pH, such as the Fenton reaction.

The generation of oxidant-free radicals by the photolytic activation of PMS could occur by two different pathways [13]. The main pathway is a consequence of the fission of an O–O bond by UV energy, according to Equation (1):

(1) $$HSO_{5}^{-}\overset{hv}{\to }SO_{4}^{-•}+HO^{•}$$

The second mechanism occurs through electron conduction. In this case, PMS is activated by an electron generated by the interaction of UV radiation with water. The mechanism follows Equations (2) and (3).

(2) $$H_{2}O\overset{hv}{\to }H^{•}+HO^{•}$$

(3) $$HSO_{5}^{-}+H^{•}\to SO_{4}^{-•}+H_{2}O$$

In both mechanisms, the oxidant-free species generated—both sulfate and hydroxyl radicals—attack the pollutants, degrading them totally or partially, according to Equation (4):

(4) $$SO_{4}^{-•}+HO^{•}+organic\;pollutants\to organic\;by-products+\;CO_{2}+H_{2}O+SO_{4}^{2-}$$

The photolytic activation of PMS has been widely reported in literature for the removal of both organic and microbiologic pollutants [7,20,21,22,23]. Some authors have focused their research on the use of UV-C radiation (λ = 254 nm), the main reason being that this wavelength provides enough energy to provoke the rupture of the O–O bond, thus generating sulfate and hydroxyl radicals to degrade pollutants. It also provokes direct photolysis of pollutants [24]. However, the use of higher wavelengths also provides high removal efficiencies. This fact has been proved by other authors, and it is confirmed in this manuscript. For instance, Pi et al. [25] reported the successful removal of sulfamonomethoxine (97%, 90 min) with UV-A radiation (365 nm) and 10 mM of PMS. Even solar radiation is able to activate PMS, reducing the cost of treatments, just like Rodríguez-Chueca et al. [26], Bandala et al. [27], or Fernández et al. [28] reported it for the elimination of organic matter, 2,4-dichlorophenol and Orange II, respectively.

**Figure 2:**
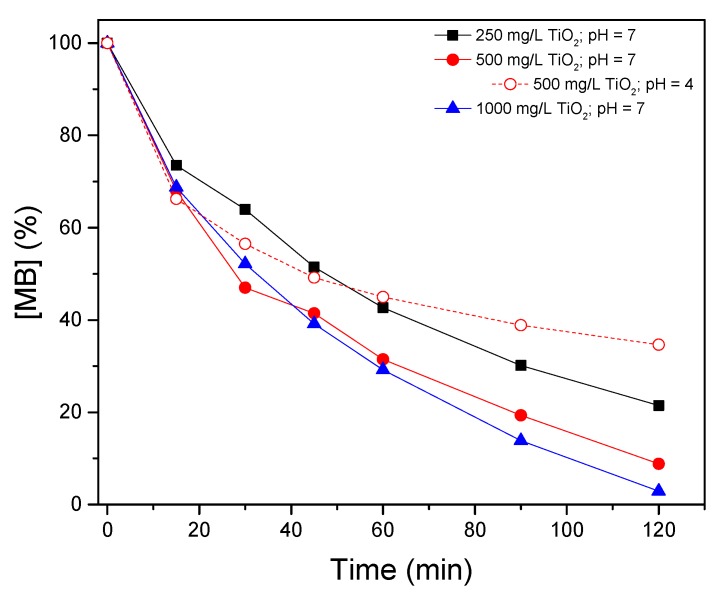
Influence of TiO_2_ dosage and pH (4 and 7) in MB discoloration through TiO_2_/UV-A.

Moreover, the mechanisms of the photocatalytic activity of TiO_2_ [29] are well-known. When photon energy greater or equal to the band gap of the semiconductor falls on the TiO_2_ surface, the in-situ generation of electrons and holes happens, since they are the promoters of active free radicals. TiO_2_ is the most used semiconductor in the treatment of water and wastewater [30]; because of its use as an activator of PMS, it is important to check its efficiency alone for the removal of MB (Figure 2). As observed in Figure 2, very small differences are obtained within the dose range studied (250–1000 mg/L). In the three cases, the removal efficiency reached higher than 80% after 120 min. Besides, the efficiency varies significantly with the variations of pH, and as can be observed in the Figure 2, treatments at pH 4 with 500 mg/L of TiO_2_ reached worse results than 250 mg/L of TiO_2_ at neutral pH.

Table 1 shows the pseudo-first-order kinetic rates for photolytic activation of PMS and TiO_2_ by UV-A radiation. Pseudo-first-order rate constants, reported as *k* (min^−1^), are estimated by linear regression fitting of the plots. As expected, the rate constants confirm the results represented in Figure 1 and Figure 2. The higher the dose of PMS or TiO_2_, the faster the discoloration of MB.

### 3.2. Photocatalytic Activation of Peroxymonosulfate by Iron Species

The activation of PMS by transition metals (Fe, Co, Ni, etc.) is widely reported in literature [15,31]. However, their users must consider possible problems of toxicity to humans and ecosystems. For that, it does not seem appropriate to use Co or Ni as an activator in the homogeneous phase, because later treatment to remove them will be required. However, iron is a more ecofriendly activator for PMS, and for that reason it was selected in this work.

**Figure 3:**
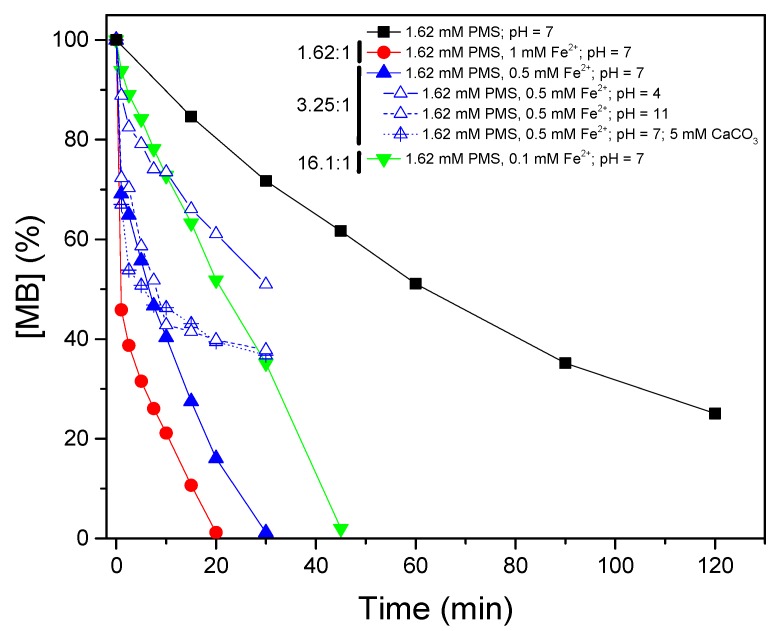
Influence of the PMS:Fe ratio (1.62:1; 3.25:1 and 16.1:1 of PMS:Fe^2+^), pH (4, 7 and 11) and the presence of scavenger substances (5 mM CaCO_3_), in MB discolouration through PMS photocatalytic activation with Fe^2+^ and UV-A radiation.

Figure 3 shows the results corresponding to the application of PMS/Fe^2+^/UV-A treatments under different conditions: different molar ratio of PMS:Fe^2+^, different pH, and the presence of CaCO_3_ as scavenger of hydroxyl radicals. Pseudo-first-order kinetic rate constants are shown in Table 2.

As can be seen in the Figure 3, very low amounts of Fe^2+^ promotes the activation of PMS, increasing the removal rate of MB. For instance, with the lowest amount of iron (0.1 mM of Fe, molar ratio PMS/Fe^2+^ = 16.2) at pH 7, total discolouration of MB was reached after 45 min of reaction. The reaction time can be reduced by increasing the Fe^2+^ concentration. For instance, the kinetic rate increased from 0.034 min^−1^ to 0.165 min^−1^, when Fe^2+^ concentration was increased 10 times (Table 2).

Fe^2+^-mediated activation of PMS occurs according the following Equations (5) and (6):

(5) $$HSO_{5}^{-}+\;Fe^{2+}\to \;SO_{4}^{•-}+Fe^{3+}+\;OH^{-}$$

(6) $$HSO_{5}^{-}+Fe^{3+}\to Fe^{2+}+SO_{5}^{•-}+H^{+}$$

The increase in the removal rate, regarding to treatments without Fe, is a consequence of the iron catalytic cycle (see a comparison of kinetic rates on Table 1 and Table 2). As observed in Equations (5) and (6), peroxymonosulfate reacts with Fe^3+^ generated by previous oxidation of Fe^2+^ with PMS, generating more oxidative species, this is the case of sulfur pentoxide radical (SO_5_^−^) and reducing Fe^3+^ to Fe^2+^, making it available again to react with PMS. However, parallel reactions (Equations (7–10)) also occur as consequence of free radicals with PMS and iron species [24].

(7) $$Fe^{2+}+SO_{4}^{•-}\to Fe^{3+}+SO_{4}^{2-}$$

(8) $$HSO_{5}^{-}+SO_{4}^{•-}\to SO_{4}^{2-}+SO_{5}^{•-}+H^{+}$$

(9) $$HSO_{5}^{-}+\;H_{2}O_{\;}\to \;SO_{4}^{2-}+2HO^{•}+H^{+}$$

(10) $$SO_{5}^{•-}+2H_{2}O\to SO_{4}^{2-}+3HO^{•}+H^{+}$$

Equations (7–10) show an excess of PMS or Fe^2+^ scavenged sulfate radicals. This fact is clearly observed in Figure 3. In this case a higher proportion of PMS is observed compared to Fe^2+^_­_, which reduces the efficiency notably. Literature shows 1:1 or 3:1 as the optimal molar ratio PMS:Fe^2+^ [32,33].

In addition, the treatment is highly affected by variations in pH. As observed in Figure 3, when a reaction is carried out at pH 4, the reaction rate decreases notably. This result agrees with the hypothesis of Sun et al., [34]. They reported that at a low pH, H^+^ scavenges sulfate and hydroxyl radicals (Equations (11) and (12)):

(11) $$HO^{•}+H^{+}+e^{-}\to H_{2}O_{\;}$$

(12) $$SO_{4}^{•-}+H^{+}+e^{-}\to HSO_{4}^{•-}$$

**Figure 4:**
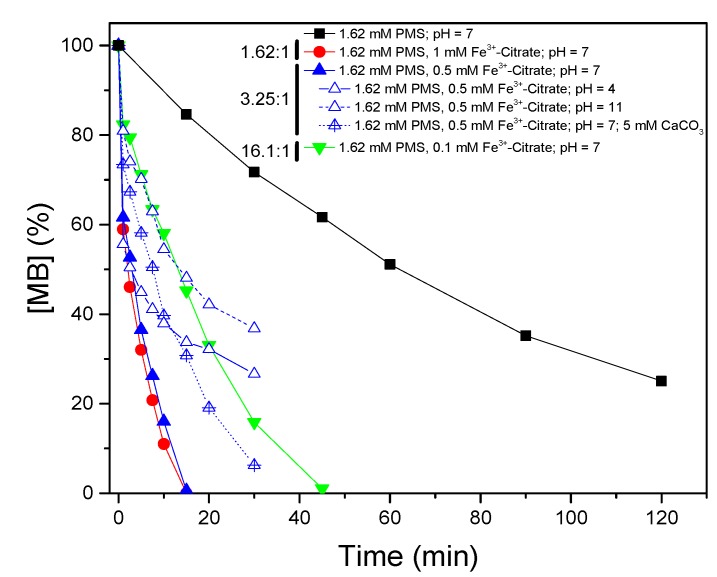
Influence of the PMS:Fe ratio (1.62:1; 3.25:1; 16.1:1), pH (4, 7, and 11) and the presence of scavenger substances (5 mM CaCO_3_) on MB decolouration through PMS photocatalytic activation with Fe^3+^-citrate and UV-A radiation.

However, the literature shows different interpretations of pH effects. Zeng et al. [35] report the highest performance of the removal of octafluorodibenzo-p-dioxin at pH 4, and the decrease of efficiency with the increase of the pH. Zeng et al. [35] related this behaviour with the precipitation of Fe^3+^ in the form of ferric oxyhydroxides at neutral or alkaline conditions, with Fe^3+^/PMS being less effective in the removal of pollutants than Fe^2+^/PMS because of the slow reduction of Fe^3+^. Other authors did not notice differences in the influence of pH on the removal of particular target compounds [15,36]. In addition, the application of this treatment at basic conditions supposes a decrease in efficiency, mainly by the formation of HCO_3_^−^/CO_3_^2−^, well-known to be a scavenger of hydroxyl radicals, which generates less reactive radicals like HCO_3_^•^ and CO_3_^•−^ [37]. The presence of CO_3_^2−^ was assessed in the efficiency of the treatment. As observed in Figure 3, the addition of 5 mM of CaCO_3_ reduces the rate significantly as a consequence of hydroxyl radical scavenging. The obtained result is quite similar to that obtained without carbonates, but at pH 11, confirming that at this pH, HCO_3_^•^ and CO_3_^•−^ can be formed. The precipitation of Fe^3+^ in the form of oxyhydroxides can be prevented using ligands, forming soluble Fe^3+^-ligand complexes. Fe^3+^-citrate is a clear example. Figure 4 shows the efficiency on MB removal by PMS/Fe^3+^-citrate/UV-A under different conditions of added doses, pH and the presence of scavengers. As expected, the addition of Fe^3+^-citrate increases the removal of MB compared to treatments without it. The comparison of PMS activation by Fe^2+^ or Fe^3+^-citrate is clearly observed with the kinetic rates shown in Table 2. The pseudo-first-order kinetic fitting shows that Fe^3+^-citrate doubles the kinetic discoloration of MB, in comparison to the use of Fe^2+^ (0.188 min^−1^ with Fe^3+^-citrate, and 0.092 min^−1^ with Fe^2+^), obtaining the total decolouration of MB after 15 min of reaction time with the molar ratio 3.25:1 PMS:Fe. The photolysis of Fe^3+^-citrate generates hydrogen peroxide (H_2_O_2_) and a superoxide radical (O_2_^•−^) (Equations (13–18)). Then, H_2_O_2_ could promote a photo-Fenton reaction with Fe^2+^ present in water from the photolysis of Fe^3+^-citrate, thus generating hydroxyl radicals (Equation (19)).

(13) $$Fe^{3+}-Cit\overset{h\upsilon }{\to }Fe^{2+}+Cit^{•}$$

(14) $$Cit^{•}\to HO^{•}CR_{2}+CO_{2}$$

(15) $$HO^{•}CR_{2}+Fe^{3+}⟶R_{2}CO+H^{+}+Fe^{2+}$$

(16) $$HO^{•}CR_{2}+O_{2}⟶R_{2}CO+H^{+}+O_{2}^{•-}$$

(17) $$2H^{+}+O_{2}^{•-}\to HO_{2}^{•}$$

(18) $$HO_{2}^{•}+HO_{2}^{•}\to H_{2}O_{2}+O_{2}$$

(19) $$H_{2}O_{2}+Fe^{2+}\to Fe^{3+}+HO^{•}+HO^{-}$$

To the best of our knowledge, the number of references in literature reporting the activation of PMS by Fe^3+^-citrate is low. For instance, Luo et al. [38] report the degradation of different organic contaminants by PMS/Fe^3+^-citrate using visible radiation by ligand-to-metal charge transfer (LMCT), while Ling et al. [39,40] used ferrous ions instead of ferric complexed with citrate to activate the PMS and remove micropollutants as carbamazepine.

However, soluble Fe^3+^-citrate avoids the generation of insoluble oxyhydroxides of Fe^3+^ at a neutral or basic pH. It can be observed in Figure 4 that the increase of pH up to 11 decreases the efficiency of the treatment slightly. The main hypothesis is that Fe^2+^ generated from the photolysis of Fe^3+^-citrate reacts again with the PMS, generating new Fe^3+^ that does not form complexes with citrate. Moreover, the addition of CaCO_3_ as a scavenger proves the presence of hydroxyl radicals, because of the deceleration of the removal of MB (0.065 min^−1^).

### 3.3. Peroxymonosulfate/TiO_2_/UV-A Synergistic Efficiency for Methylene Blue Removal

**Figure 5:**
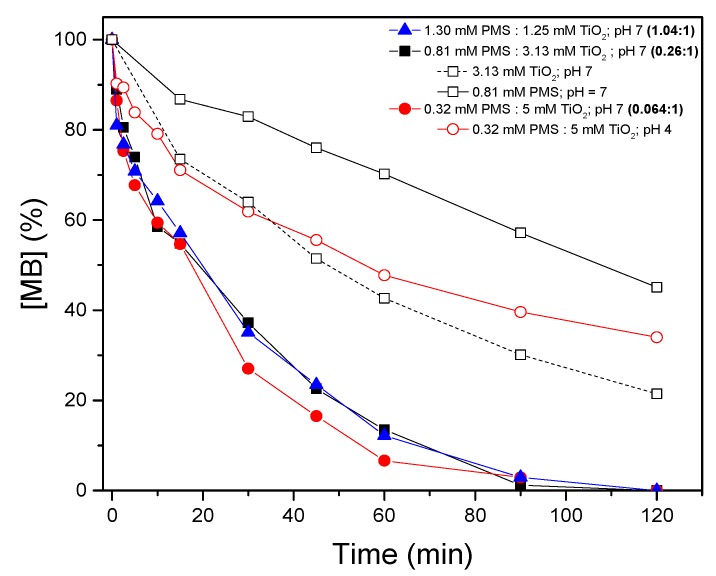
Influence of the PMS:TiO_2_ ratio (1.04:1; 0.26:1; 0.064:1) and pH (4 and 7) in MB discolouration through PMS photocatalytic activation with TiO_2_ and UV-A radiation.

After the successful activation of PMS by Fe^2+^ and Fe^3+^-citrate, this research explores the use of TiO_2_ as an activator. Both PMS and TiO_2_ generate free oxidant radicals in the presence of UV radiation, as shown in Figure 1 and Figure 2, so the combination of PMS and TiO_2_ was added in lower dosages than if applied individually. The results are represented in Figure 5, and Table 3 shows the kinetic rate constants.

As observed in Figure 5, different molar ratios of PMS:TiO_2_ were tested (1.04:1, 0.26:1 and 0.064:1). The three different combinations of PMS:TiO_2_ enhanced the efficiency of PMS and TiO_2_ individually, the final removal of MB was quite similar in all these combinations. Under these conditions, total removal of MB was reached after 90 min of treatment. The efficiency is lower than that obtained in iron-mediated activation of PMS, but it has to be remarked that dosages of reagents have been reduced in this case. This enhancement is a consequence of PMS activation (sulfate and hydroxyl radical generation), because of the reaction with the free electron on the surface of TiO_2_, according to Equations (20) and (21) [18].

(20) $$HSO_{5}^{-}+e_{CB}^{-}\to SO_{4}^{•-}+HO^{-}$$

(21) $$HSO_{5}^{-}+e_{CB}^{-}\to SO_{4}^{2-}+HO^{•}$$

**Scheme 1:**
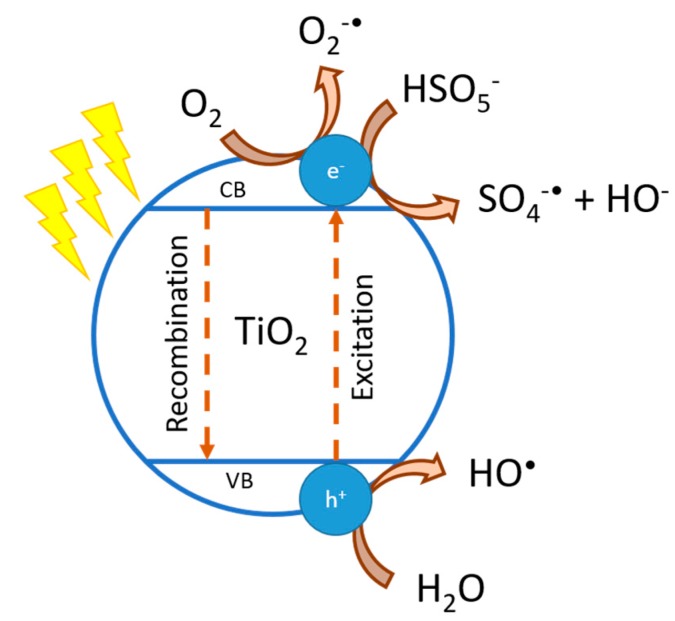
Generation of sulfate and hydroxyl radicals by PMS/TiO_2_/UV-A treatments.

Scheme 1 describes the reaction occurring by a combination of PMS/TiO_2_/UV-A radiation.

An evaluation of the synergistic effects when combining activation factors is made using rate constants to calculate a synergistic factor [14,41] (Equation (22)):

(22) $$S_{PMS-TiO_{2}-UVA}=\frac{k_{PMS-TiO_{2}-UVA}}{k_{PMS-UVA}+k_{TiO_{2}-UVA}}$$

Table 4 shows the constant rates and the synergistic factor for PMS activation by TiO_2_. A synergistic factor of *S_PMS/TiO2/UVA_* = 1.79 is estimated. This value suggests that synergy is occurring. Although this synergistic factor is not so high, it verifies the activation of PMS by TiO_2_.

The references in literature regarding the activation of PMS with TiO_2_ are scarce. Golshan et al. [16] synthesise a catalyst based on TiO_2_ and copper ferrite that is able to activate PMS in a greater way than TiO_2_ alone, in order to degrade 2,4-dichlorophenoxyacetic acid. On the other hand, Jo et al. [17] reported the successful activation of PMS on TiO_2_ via a ligand-to-metal charge transfer using visible light (λ > 420 nm). The main hypothesis is the generation of a visible light-absorbing complex on the TiO_2_ surface, and the visible light-induced LMCT that leads to electron injection into the CB (conduction band) of TiO_2_. However, other authors reported the activation using UV-A and UV-C radiation. For instance, Zazouli et al. [19] reported the use of different UV sources for the activation of PMS with a catalyst composed of Fe_3_O_4_–TiO_2_ on the removal of the Brilliant Blue FCF dye.

## 4. Conclusions

This work was focused on the different activation routes of peroxymonosulfate to remove organic pollutants in water. The conclusions obtained can be summarized as follows:

- Low dosages of PMS (0.81–3.25 mM) are enough to remove organic pollutants like MB. Activation of PMS by UV-A radiation increases the removal rate, reducing the contact time to reach complete removal of pollutants.
- The pH plays an important role. Neutral conditions reached the best efficiencies, while efficiency was reduced at acidic (pH 4) and basic (pH 11) conditions as a consequence of scavenger reactions.
- Photocatalytic activation of PMS with iron species accelerates the generation of sulfate radicals and therefore the MB removal. The molar ratio of PMS:Fe is crucial, because an excess of PMS scavenges the oxidant radicals.
- The use of Fe^3+^-citrate as a catalyst showed the best performance, reaching total removal of MB in 15 min of reaction. The reason is related to the generation of hydrogen peroxide (H_2_O_2_) and superoxide radical (O_2_^−^) by the photolysis of Fe^3+^-citrate. In addition, the variations in pH do not affect the reaction because of the complexation of Fe^3+^, which avoids the formation of oxyhydroxides of Fe^3+^.
- PMS is successfully activated by TiO_2_, reaching the total removal of MB in 90 min. The PMS activation occurs because of the reaction with the free electron on the surface of TiO_2_, where there is no influence of the molar ratio of PMS:TiO_2_ (1:04:1; 0.26:1; 0.064:1).

## Figures and Tables

**Table 1:** Pseudo-first-order kinetic rates (min^−1^) for the photolytic activation of PMS and TiO_2_.

Experimental Conditions	pH	Rate Constant (min^−1^)	*R* ^2^
0.81 mM PMS/UV-A	7	0.006	0.99
1.62 mM PMS/UV-A	7	0.011	0.99
1.62 mM PMS/UV-A	4	0.008	0.99
1.62 mM PMS/UV-A	11	0.009	0.99
3.25 mM PMS/UV-A	7	0.018	0.98
3.16 mM TiO_2_/UV-A	7	0.013	0.98
3.16 mM TiO_2_/UV-A	4	0.011	0.67
6.26 mM TiO_2_/UV-A	7	0.020	0.98
12.52 mM TiO_2_/UV-A	7	0.025	0.94

**Table 2:** Pseudo-first-order kinetic rates (min^−1^) for the photocatalytic activation of PMS with Fe^2+^ and Fe^3+^-citrate under different operating conditions.

Experimental Conditions	pH	Rate Constant (min^−1^)	*R* ^2^
1.62 mM PMS/1 mM Fe^2+^/UV-A	7	0.165	0.70
1.62 mM PMS/0.5 mM Fe^2+^/UV-A	7	0.092	0.94
1.62 mM PMS/0.5 mM Fe^2+^/UV-A + 5 mM CaCO_3_	7	0.092	0.82
1.62 mM PMS/0.5 mM Fe^2+^/UV-A	4	N.F.	
1.62 mM PMS/0.5 mM Fe^2+^/UV-A	11	0.025	0.82
1.62 mM PMS/0.1 mM Fe^2+^/UV-A	7	0.034	0.99
1.62 mM PMS/1 mM Fe^3+^-Citrate/UV-A	7	0.223	0.95
1.62 mM PMS/0.5 mM Fe^3+^-Citrate /UV-A	7	0.188	0.94
1.62 mM PMS/0.5 mM Fe^3+^-Citrate /UV-A + 5 mM CaCO_3_	7	0.065	0.80
1.62 mM PMS/0.5 mM Fe^3+^-Citrate /UV-A	4	0.088	0.98
1.62 mM PMS/0.5 mM Fe^3+^-Citrate /UV-A	11	N.F.	
1.62 mM PMS/0.1 mM Fe^3+^-Citrate /UV-A	7	0.059	0.98

**Table 3:** First-order kinetic rates (min^−1^) for the photocatalytic activation of PMS with TiO_2_ under different operating conditions.

Experimental Conditions	pH	Rate Constant (min^−1^)	*R* ^2^
1.30 mM PMS/1.25 mM TiO_2_/UV-A	7	0.035	0.97
0.81 mM PMS/3.16 mM TiO_2_/UV-A	7	0.034	0.97
0.32 mM PMS/5 mM TiO_2_/UV-A	7	0.044	0.98
0.32 mM PMS/5 mM TiO_2_/UV-A	4	0.014	0.87

**Table 4:** Synergistic factor for PMS activation by TiO_2_ and UV-A radiation.

Experiment	Experimental Conditions	First-Order Rate Constant (min^−1^)	Synergistic Factor
1	0.81 mM PMS/UV-A	0.006	1.79
2	3.16 mM TiO_2_/UV-A	0.013	
3	0.81:3.16 mM PMS:TiO_2_/UV-A	0.034	
